# Multiphoton microscopy at a microwatt level via gain-managed nonlinear amplification and pulse-picking

**DOI:** 10.1364/BOE.557132

**Published:** 2025-03-27

**Authors:** Katarzyna Kunio, Grzegorz Soboń, Jakub Bogusławski

**Affiliations:** Laser & Fiber Electronics Group, Faculty of Electronics, Photonics and Microsystems, Wrocław University of Science and Technology, Wybrzeże Wyspiańskiego 27, 50-370 Wrocław, Poland

## Abstract

We introduce a compact, all-fiber laser system with a gain-managed nonlinear (GMN) amplified Yb:fiber oscillator and an integrated pulse-picker. The system delivers 39 fs pulses with peak powers of 0.83 MW and adjustable pulse repetition rates (0.3–15 MHz), enabling multiphoton imaging at remarkably low excitation powers (as low as 66 µW). Its design simplifies integration and enhances experimental flexibility. Compatible with two- and three-photon excitation, but also second harmonic generation microscopy, this versatile system offers precise control of imaging parameters, making it an effective tool for advancing multiphoton microscopy and other imaging techniques across various experimental environments.

## 1. Introduction

Multiphoton microscopy is an advanced, high-resolution imaging method that utilizes near-infrared light to visualize biological structures deep within tissues [1–3]. The multiphoton process occurs only when multiple photons interact simultaneously, which requires high photon density, usually found at the focal point of an objective. Because of this, excitation is confined to a specific volume. Additionally, the use of long, near-infrared wavelengths minimizes damage to the tissue. This characteristic makes it particularly useful for in vivo imaging and long-term cellular dynamics studies in intact organisms [4,5]. Multiphoton microscopy has been instrumental in many fields; for example, it can be used in studying embryonic development [6,7] and observing cancer progression [8,9] in biomedical research. It can also be employed in examining the microstructures in material science [10,11], and investigating plant structures in environmental science [12,13]. The potential for non-invasive imaging of delicate tissues has been confirmed through its application in ophthalmology, enabling in vivo imaging of the retina in both mice and humans [14,15].

Multiphoton microscopy commonly employs femtosecond pulsed lasers, e.g., Ti:Sapphire lasers [16–18], because of their ability to generate high peak intensities required for multiphoton excitation. These lasers also offer a broad tunability (approximately 700–1050 nm) in the near-infrared range. However, they are expensive, bulky, and need careful alignment and maintenance, which may make their operation complex.

Emerging alternatives, like fiber lasers, overcome those drawbacks but often have a narrower tuning range and lower peak power, limiting their versatility [19]. The output pulses from a fiber oscillator can be amplified to increase power; however, in the case of Yb-doped fibers, the pulse duration is limited to ∼120 fs due to gain narrowing, which also imposes a constraint on the achievable peak power [20–22]. These disadvantages could be resolved using a GMN amplifier [23–26]. In a GMN amplifier, the pulse is amplified within the active medium, where nonlinear effects like self-phase modulation are managed by carefully choosing the medium’s length and the pump power. The careful balance between these parameters prevents excessive pulse distortion from nonlinear phase accumulation and allows substantial spectral broadening (facilitating sub-100 fs pulse generation or wavelength tunability). Consequently, the output spectrum of a GMN amplifier becomes significantly broader than the input, typically displaying a smooth shape and linear chirp that allow for efficient compression into ultrashort pulses. This method generates high-energy pulses with extremely short durations and maintains excellent spectral coherence, making it very attractive for application in multiphoton microscopy. Previous studies successfully demonstrated multiphoton microscopy with a GMN amplifier [27], however, the system contained multiple bulk-optics components, making it prone to misalignment and more challenging to operate due to the increased complexity.

While GMN amplification allows one to obtain ultrashort pulses which improves image quality in multiphoton microscopy, other pulse train parameters are important as well. The intensity of the fluorescence signal obtained during the imaging depends on the relationship between the square of the average power (*P_avg_*) at the sample, the duration of the pulse (*τ_p_*, and consequently the peak power, *P_peak_*), and the repetition frequency (*f_rep_*) of the pulse train:

(1) $$n∼\frac{P_{avg}^{2}}{\tau_{p}\cdot f_{rep}}=P_{peak}^{2}\cdot \tau_{p}\cdot f_{rep},$$

where *n* is the average number of photons emitted by the fluorescing medium per second. It is possible to maintain the signal value while lowering the average power of the excitation beam by proportionally decreasing the repetition frequency, which translates to increased pulse peak power and energy. This effect can be achieved by adding the pulse picker unit to the laser system [28,29] and is particularly advantageous in applications where maintaining low average power at the sample is essential. Examples include live cell imaging, such as neuronal imaging in brain tissues (where the functional integrity must be preserved) [30], embryonic development studies (to prevent disruption of sensitive biological processes) [6,31], and long-term time-lapse imaging of dynamic cellular behaviors [32]. Other examples include skin and ophthalmic imaging; it was shown that the primary damage mechanism is of thermal origin and is caused by one-photon absorption of infrared light by melanin granules [14,33]. The ability to regulate the excitation power while preserving high pulse energy ensures flexibility and precision in achieving optimal imaging conditions for diverse applications.

Here, for the first time, we demonstrate multiphoton microscopy conducted using the all-fiber GMN amplification of Yb:fiber oscillator with an integrated pulse picker unit. The laser generated pulses as short as 39 fs with peak powers of 0.83 MW and features an adjustable *f_rep_* within the 0.3–15 MHz range. Such a combination allows for efficient multiphoton imaging at a very low average excitation power. We evaluated the applicability of the laser system in multiphoton microscopy in various modalities, including two-photon excited fluorescence (2PEF), second-harmonic generation (SHG), and three-photon excited fluorescence (3PEF).

## 2. Experimental setup and methods

**Fig. 1.**
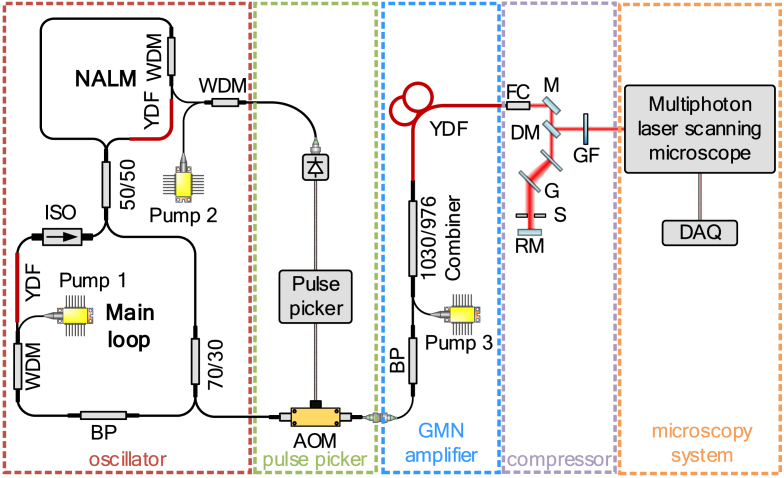
Experimental setup. BP – bandpass filter, YDF – Yb-doped fiber, WDM – wavelength-division multiplier, ISO – isolator, AOM – acousto-optic modulator, FC – fiber collimator, M – mirror, DM – D-shaped mirror, G – diffraction grating, S – slit, RM – retro mirror, GF – gradient index filter, DAQ – data acquisition card.

The experimental setup is shown in Fig. 1. It consisted of five major units: oscillator, pulse picker, GMN amplifier, pulse compressor, and multiphoton microscope. The first three units were fully fiber-optic and utilized only polarization-maintaining fibers.

**Fig. 2.**
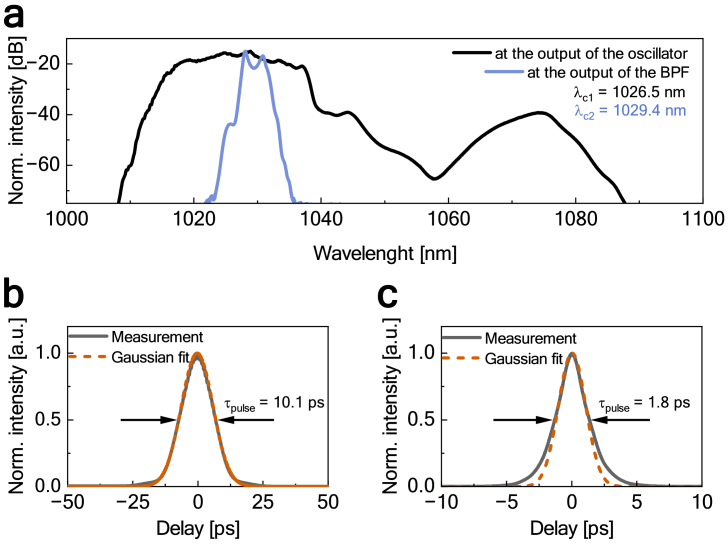
Characterization of the pulse: (a) optical spectrum at the output of the oscillator (black) and at the output of the BP (blue), (b) autocorrelation with the Gaussian fit at the output of the oscillator, (c) autocorrelation with the Gaussian fit at the output of the BP.

We have employed a self-made Yb-doped oscillator in a figure-eight configuration, characterized in detail in [28]. It consisted of two loops: the main loop and the nonlinear amplification loop mirror (NALM). The oscillator produced 10 ps pulses at the central wavelength of 1026.5 nm [Fig. 2(a)-(b)] with ∼10 nJ energy and a repetition frequency of 15.2 MHz. The smaller peak at around 1075 nm was caused by Raman scattering (characteristic shift of 440 cm^−1^ for fused silica fibers [34]). The oscillator had excellent long-term power stability; root-mean-square (RMS) power stability was at the level of 0.05% (over 3 h).

The output of the oscillator was then connected to the pulse picking unit which allows the user to manipulate the repetition rate of the system by dividing its value by a positive integer. It consisted of an acousto-optic modulator (AOM, G&H Fiber-Q) and an electronic driver (AA Optoelectronic PPKS200). The operation of the pulse picking unit was synchronized with the pulse train provided by the oscillator to the photodiode (Thorlabs PDA05CF2) using one of the unused ports of the wavelength-division multiplexer (WDM).

Subsequently, the output of the AOM was connected to the GMN amplifier. Before entering the amplifier, the pulse passed through a 2-nm bandpass filter (BP) to bring its duration closer to the transform limit. The BP filter narrowed the spectrum and reduced the pulse duration to approx. 1.8 ps, as illustrated in Fig. 2(c). Although the Gaussian fit does not perfectly match the pulse shape, likely due to higher-order dispersion of the filter causing slight chirping [35], it is used here to provide a rough estimate of the pulse’s FWHM duration. Comparing the time-bandwidth product values for the pulse before and after propagation through the BP filter (44.8 and 1.68, respectively), it is clear that the pulse was much closer to the transform limit. Usually, this effect is achieved by a bulk-optic compressor placed between the oscillator and the GMN amplifier [25,26]. Our use of the BP filter makes the setup more compact (as we only use fiber elements) and easier to use.

Using the 976/1030 pump/signal combiner, we added the 10 W multimode 976 nm diode to our system to pump the double-clad Yb-doped fiber (Nufern PLMA-YDF-10/125-VIII) of a length of 3.6 meters. The characterization of the pulse train at the amplifier’s output is shown in the Supplement 1. The output pulse was then compressed using a Treacy compressor [36] consisting of two parallel transmission diffraction gratings (Coherent LightSmyth, 1000 grooves/mm, G) with an estimated group delay dispersion of -0.016 ps^2^. The transmission of the compressor was ∼80%. After transmission through both diffraction gratings, the pulse traveled through the slit (S) placed there to block the part of the spectrum generated by Raman scattering.

**Fig. 3.**
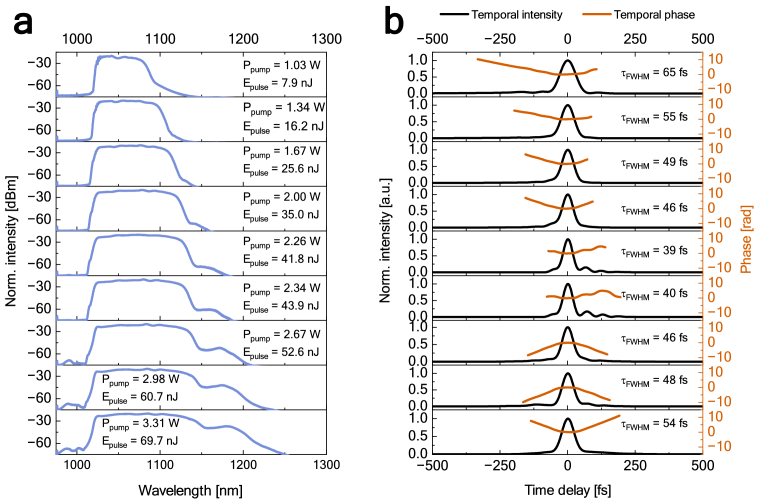
Characterization of the pulse after amplification and compression at different values of the pump power measured at the fundamental repetition frequency of 15.2 MHz: (a) optical spectra and (b) FROG-retrieved temporal intensity (black) with temporal phase (red). The temporal phase was plotted at >2% temporal intensity.

The optical pulse spectra at different pump power values after propagation through the compressor are shown in Fig. 3(a). It can be seen that the optical bandwidth of the spectrum and the pulse energy increased with the increase in pump power, from a bandwidth of 45.8 nm and energy of 7.9 nJ at the lowest measured pump power of 1.03 W to the bandwidth of 100.9 nm and energy of 69.7 nJ at the highest pump power of 3.31 W. Consequently, the central wavelength gradually moved towards longer wavelengths, from 1048.4 nm at 1.03 W pump power to 1074.9 nm at 3.31 W pump power. All values of the 3 dB optical bandwidth and central wavelength at different pump power values are shown in Tab. 1. The smaller peak noticeable at longer wavelengths is caused by Raman scattering, and its intensity and bandwidth also increased with the increase in pump power.

**Table 1.** Central wavelengths and optical bandwidths of the optical spectra at different pump power values.

Pump power [W]	λc [nm]	Δλ [nm]
1.03	1048.4	45.8
1.34	1058.6	65.1
1.67	1063.1	74.9
2.00	1067.1	82.5
2.26	1070.5	89.3
2.34	1071.0	86.7
2.67	1075.6	89.5
2.98	1077.0	89.8
3.31	1074.9	100.9

The temporal profile of the pulse after compression was characterized using SHG frequency-resolved optical gating (FROG). For this purpose, we used a commercially available FROGscan Ultra2 (Mesa Photonics Inc.). We note that SHG FROG traces are symmetric with respect to time delay, creating temporal ambiguity, so a pulse and its time-reversed version produce identical traces. As a result, positively and negatively chirped pulses are indistinguishable [37–39]. Figure 3(b) shows those profiles at selected pump powers, along with the temporal phase. The pulse duration gradually decreased with the increase in pump power and plateaued at 2.26 W, from which point it started increasing.

**Fig. 4.**
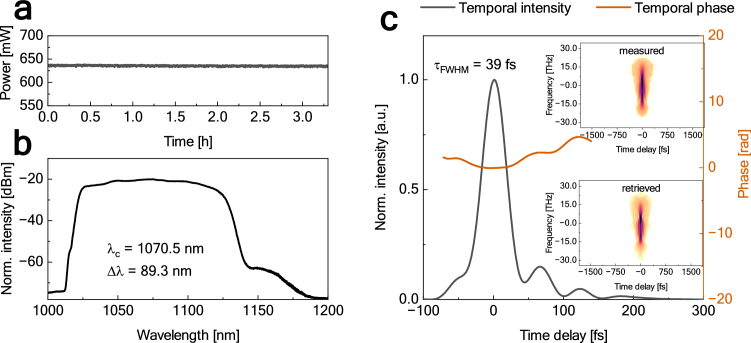
(a) The long-term power stability measurement of the pulse at the compressor's output at the fundamental frequency of 15.23 MHz. Characterization of the pulse at the pump power of 2.26 W: (b) optical spectrum, (c) FROG-retrieved temporal intensity (black) with temporal phase (red).

The shortest pulse duration of 39 fs was achieved at the pump power value of 2.26 W and the fundamental repetition frequency of 15.2 MHz. The output power at this configuration was equal to 635.62 mW, corresponding to the pulse energy of 41.82 nJ and peak power of 0.83 MW. The long-term power stability measurement of the pulse at the output of the compressor is shown in Fig. 4(a) and it displays an excellent power stability of 0.16% RMS over 3 h (after a 1 h warm-up time). Figures 4(b)-(c) show a closer characterization of this pulse. The optical spectrum was centered at 1070.5 nm with a 3 dB optical bandwidth of 89.3 nm. The shape of the temporal phase is relatively flat.

Using the pulse picker, it was possible to reduce the *f_rep_* of the system. In such a case, reducing the pump power was required to maintain the same nonlinear amplification process for each pulse and reach the same pulse duration and peak power after the compression. We note that pulse compression was slightly less efficient at lower repetition rates. For example, it was possible to compress the pulse to 41 fs at 1.52 MHz, and 52 fs at 0.3 MHz. In our system, the optimal pulse compression is achieved by adjusting the pump power for different *f_rep_*, while keeping the pulse compressor settings fixed. At fundamental *f_rep_*, the best compression occurs at 2.26 W pump power, but lower rates require reduced power. However, precise adjustments at low power levels are difficult due to the limited 10 mA resolution of the laser diode controller, reducing compression efficiency at lower *f_rep_*. This could be resolved with, e.g., a more precise laser diode driver.

## 3. Results

The compressed pulse was guided into the custom-built multiphoton scanning microscope (described in detail in [29]). The system is versatile and compatible with multiple imaging modalities, enabling operation with techniques such as 2PEF, 3PEF, SHG, and third harmonic generation (THG). The light emitted by the sample was detected in epi-geometry using a photomultiplier tube (PMT, Thorlabs PMT2101 with Hamamatsu H10770PA-40 GaAsP photocathode). The PMT signal was filtered with a 100 kHz low-pass filter and digitized using a data acquisition card (DAQ, NI PCIe-6363). A 400–700 nm bandpass filter was placed in front of the PMT. A custom-written NI LabVIEW software was used to control the system. Each registered image was 512 × 512 pixels, and the pixel dwell time was set to 5 µs. A gradient index filter (GF, Thorlabs NDL-25C-2) at the entrance to the microscope was used to adjust the average power guided to the sample. The position of the diffraction grating in the compressor was adjusted to compensate for the dispersion introduced by the optical components of the microscope, allowing us to use as short a pulse as possible directly in the sample plane. The compressed pulse was used in all further experiments.

### 3.1. Imaging at low average powers

The first experiment aimed to lower the average power used during imaging as much as possible. To do so, we lowered the repetition frequency of the pulse train, which naturally reduced the average power at the sample and, consequently, the fluorescence signal. However, we note that we only used a fraction of the available average power. By adjusting the gradient filter at the input of the microscope, we were able to increase the average power while maintaining the reduced repetition rate, compensating for the loss in fluorescence signal and ensuring the same amount of nonlinear response for optimal imaging due to the increased pulse energy.

**Fig. 5.**
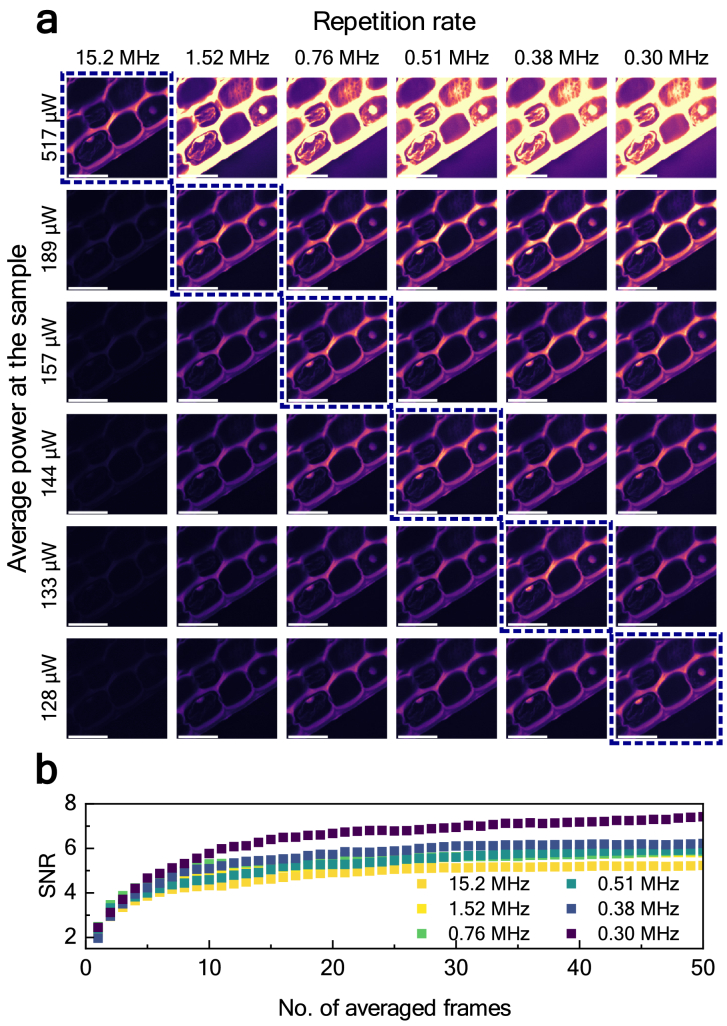
Analysis of the influence of lowering both the repetition frequency and average power at the sample on 2PEF microscopy: (a) 2PEF images of *convallaria majalis* obtained by gradually decreasing the repetition frequency while simultaneously decreasing the average power at the sample. Dashed blue lines mark images with the same average signal per frame. Scale bars: 80 µm. (b) Change in the SNR depending on the repetition frequency [for images at the diagonal of the panel (a)].

For this experiment we used a sample of *convallaria majalis* root transverse section stained with an acridine orange. According to Eq. (1), we could maintain the level of the mean signal received during imaging by simultaneously reducing both the repetition frequency and the average power at the sample. The fundamental frequency of 15.2 MHz (already 5 times lower than the 80 MHz typically used in Ti:Sapphire lasers [40,41]) was reduced up to 50 times, resulting in the minimal repetition frequency of 0.3 MHz. Figure 5(a) shows the results of imaging *convallaria majalis* at different repetition frequencies and average powers. All presented results were received after averaging 50 frames. Examining the results at the fundamental repetition frequency of 15.2 MHz reveals that as the average power at the sample decreases, the signal drops significantly, resulting in a substantial loss of information. Conversely, maintaining the initial average power of 517 µW while reducing the repetition frequency quickly causes the PMT to saturate. Finally, looking at a diagonal, it can be noticed that we managed to decrease the average power four times (517 µW at the fundamental frequency compared to 128 µW at 0.3 MHz) while maintaining the same value of the mean fluorescence signal due to the increased pulse peak power (for details see Dataset 1 [42]). Theoretically, it should be possible to reduce the average power $\sqrt{50}=7$ times, i.e., to 74 µW. The fact that higher power was necessary may result from slightly longer pulse duration at low repetition rate. The experiment shows that this method allows for a significant reduction in the average power needed for successful imaging.

Figure 5(b) shows the change in the signal-to-noise ratio (SNR) [43] value during the averaging process for different values of repetition frequency. Regions of interest used to calculate the SNR were chosen in the brightest part of the image (signal) and outside the sample (noise, bottom right). After averaging around 10 frames, it becomes clear that the lower the frequency of repetition, the higher the SNR. While the mean value of the signal maintains the same value, both the mean value and the standard deviation of the background signal decrease with the decrease in the repetition frequency (thus increasing the SNR). This drop in the value of the mean background signal could be attributed to reduced scattering within the microscope system or the microscope slide, resulting from the lower average power delivered to the sample. This experiment showed that reducing the repetition rate allows for imaging at lower average power and with higher SNR. Specifically, reducing the *f_rep_* by 50 times allowed us to reduce the average power four times and improve the SNR by 42%.

**Fig. 6.**
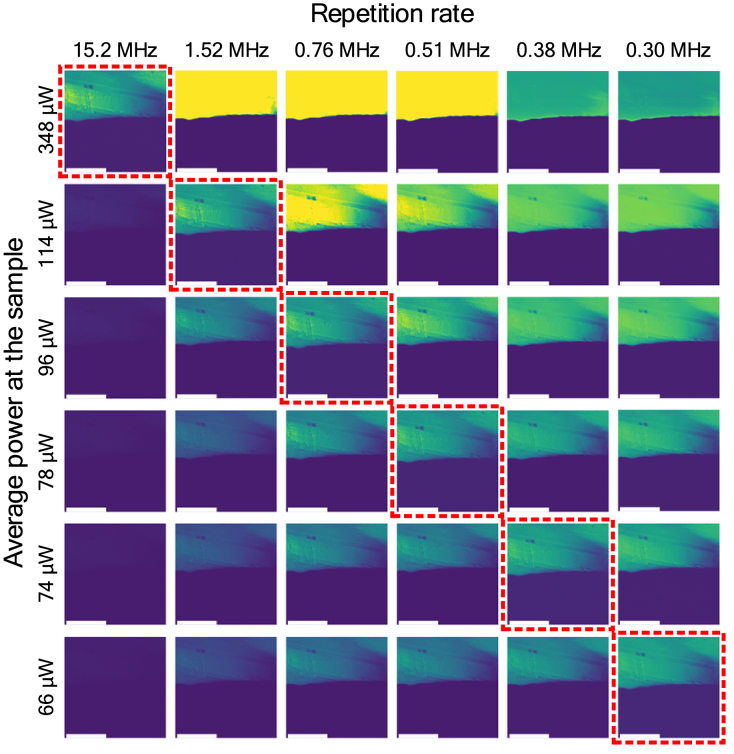
SHG microscopy images of urea microcrystals obtained by gradually decreasing the repetition frequency while simultaneously decreasing the average power at the sample. Scale bars: 80 µm.

Next, we performed a similar experiment in a different imaging modality, i.e., SHG microscopy using urea microcrystals as a sample. These results are shown in Fig. 6. Similarly to the previous case, we were able to significantly reduce the average power needed to obtain quality images by simultaneously reducing the repetition rate. This sample had a greater response to our pulses, as only the maximum of 348 µW of average power at the sample was needed to receive good images using pulses at the fundamental frequency. We also managed to decrease this value five times (to 66 µW) to receive the same image at the lowest repetition frequency of 0.3 MHz. Moreover, we noticed that with each decrease in repetition frequency and average power structural details of the sample started to vanish, suggesting the PMT saturation.

**Fig. 7.**
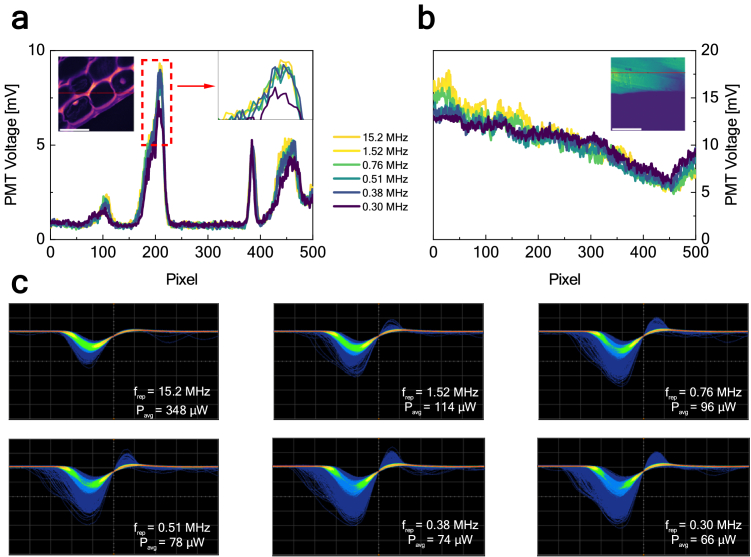
Comparison of pixel intensities in the images obtained at the same values of the mean signal and different values of the repetition frequency measured in (a) row 250 of images depicting *convallaria majalis* and (b) row 110 of images depicting urea microcrystals. Scale bars: 80 µm. (c) Oscilloscope traces of the detector response measured while imaging urea microcrystals at different repetition frequencies.

To investigate this effect further, for both samples, we checked the PMT voltage in one row of the images obtained at different values of the repetition frequency and average power but the same value of the mean signal (in both cases, we refer here to the images marked with the dashed lines, the diagonal). Results for *convallaria majalis* and urea microcrystals are shown in Fig. 7(a)–(b). Despite the overall mean signal being approximately the same, we have noticed its decrease in parts of the image producing the highest signal, e.g., the first 200 pixels in the observed pixel row in Fig. 7(b). The signal collected while imaging using the lowest repetition frequency is visibly lower than at higher repetition frequencies. Some information is, therefore, lost, as the signal is flat.

To further investigate this effect, we directly recorded oscilloscope traces of the PMT response to a single excitation pulse (using Agilent Infiniium DSO90604, bandwidth 6 GHz, 20 GS/s; the 100 kHz low-pass filter was removed for this experiment). Here, urea microcrystals were used as a sample; we used the same combination of average power and repetition frequency, which resulted in images with similar mean intensity per frame. We measured around 30000 PMT pulses for each configuration, and the presented results show a heatmap signifying how often the pulse kept a certain shape. This allows us to examine dynamic differences in PMT response to excitation pulses with different peak powers. At the fundamental frequency, the pulse kept a uniform and more repeatable shape, however, with each decrease of the repetition frequency and average power at the sample, the pulse began to show more and more distortions, characteristic of the effect of PMT saturation [44].

### 3.2. Hyperspectral imaging by the spectral phasor approach using sine and cosine filters

Our results demonstrated that it is possible to obtain high-quality images at a very low excitation average power. To further demonstrate the usability of such imaging conditions, we performed hyperspectral imaging via the spectral phasor approach [45,46]. This approach is a well-established method for data visualization and image analysis in spectral fluorescence microscopy. The basic idea is that it is not necessary to use the full spectrum for certain classical spectral analysis methods, such as demixing. Instead, only a few parameters are often sufficient, such as the center of a mass and the bandwidth of the spectrum, and the first component of the spectral Fourier transform can be used as a proxy for the entire spectrum. A common approach to hyperspectral microscopy is to record fluorescence emission with a spectrograph to provide the spectrum for each pixel, which is subsequently phasor transformed and described with two parameters G and S [45,46].

**Fig. 8.**
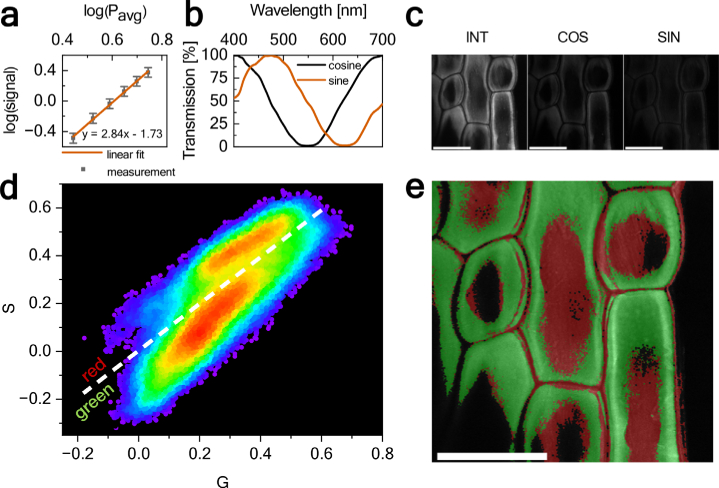
(a) Power-dependent fluorescence intensity recorded in the outer epidermal cell layer. Spectral phasor-based 3PEF imaging of epidermal cells without staining: (b) transmission spectra of sine and cosine filters, (c) epidermal cell images obtained without an additional filter in front of the PMT (INT), with cosine (COS), and with sine (SIN) filters, (d) spectral phasor plot (white dashed line indicates the result of GMM clustering), and (e) spectral phasor-based color-coded image of epidermal cells. Scale bars: 200 µm.

For this experiment, we chose a 3PEF modality and an unstained sample, which typically requires the use of a laser with a high average power. Three-photon excitation at 1070.5 nm central wavelength corresponds to single-photon excitation at ∼357 nm, i.e., it is well-suited for the excitation of endogenous fluorophores in plants [47]. As an example, we imaged the outer epidermal cell layer (extracted from an onion). A 580 µW average excitation power and 0.3 MHz repetition rate were used. A three-photon process was confirmed by the cubic relationship between the excitation power and fluorescence intensity [Fig. 8(a)].

To obtain spectral information, phasor transform was performed ‘in hardware’ using two, specifically designed filters placed in front of the PMT instead of a spectrograph [48,49]. Dvornikov and Gratton demonstrated that the transformation can be performed using simple plastic filters and correction for the non-ideal response of the filters can be introduced in post-processing. In contrast, here we used custom-designed filters with transmission characteristics almost perfectly resembling the sine and cosine function over the 400 - 700 nm range [Fig. 8(b)]. Within this range, filters feature one period of each function, therefore, they can be used to obtain the first harmonic of the spectral bandwidth. Both filters were custom-designed and fabricated by OptoSigma (170926DD01 and 170926DD02). Outside the 400 - 700 nm range, the transmission of the system is zero due to the use of a bandpass filter (Thorlabs FESH0700). This allows us to skip the correction stage for the non-ideal response of the filters. In this approach, it is necessary to record a set of three images of the sample: without any additional filters (INT), with cosine (COS), and with sine (SIN) filters [Fig. 8(c)]. Using those images and following the procedure outlined in Ref. [48], it is possible to calculate the spectral phasor plot, shown in Fig. 8(d). Two distinctive phasor groups are visible in the plot, indicating the presence of pixels with two different spectral characteristics. Similar to Ref. [50], we used the Gaussian mixture model (GMM) for clustering and color-coding pixels in the image (implemented in MATLAB, using a built-in function); two clusters were assumed. The result of clustering is indicated with a white dashed line, separating phasor points into two groups. Finally, those groups were color-coded and marked in the original image, as shown in Fig. 8(e), where epidermal cells as well as cellulose fibrils are visible [51]. The first cluster (green in panel e), containing a larger number of pixels in the image, was centered around 424 nm with 214 nm bandwidth. The second cluster (red) was centered around 442 nm with 151 nm bandwidth. Blue fluorescence emission with broad spectra is typical for UV-excited plant autofluorescence [47].

## 4. Discussion

This study presented a novel all-fiber, all-polarization maintaining laser setup incorporating a GMN amplifier and demonstrated its potential for advanced imaging applications. While using a GMN amplifier in multiphoton microscopy was previously shown in only one study [27], our work significantly advanced the field by introducing a fully fiber-integrated system up until the output of the GMN amplifier. This design offers several advantages, including compactness and ease of alignment compared to free-space systems, making it more robust and practical for use in diverse experimental environments. Additionally, integration of the pulse picking unit in our setup enabled control over the laser’s repetition rate, allowing for imaging to be performed at very low average power levels (even as low as 66 µW). Low-power imaging is particularly advantageous for applications requiring extended observation of living specimens [4,5,52,53], where photodamage and photobleaching must be minimized to preserve sample viability. Other scenarios where average power has to be minimized to avoid thermal damage are skin and ophthalmic imaging [14,33]. Furthermore, it is beneficial for imaging fragile materials or structures with low damage thresholds, such as thin films, microelectronic, or nanoscale devices [10,11]. These innovations position our system as a significant improvement over existing approaches, with a broad potential for use in multiphoton microscopy and other imaging modalities.

The incorporation of the GMN amplifier in our setup makes it universal for use in many multiphoton modalities, such as 2PEF and 3PEF, but also SHG and THG. It is particularly well-suited for three-photon microscopy. The GMN amplifier enables the use of longer wavelengths, such as our central wavelength of around 1070 nm, which is critical for efficient 3PEF. Longer wavelengths reduce scattering within biological tissues, enhance penetration depth, and mitigate photodamage, ensuring safer and more effective imaging. The high energy output provided by the GMN amplifier is another significant advantage, as it facilitates a robust 3PEF and generates sufficient signal even in challenging imaging conditions. Notably, at our effective absorption wavelength of ∼350 nm (achievable through three-photon excitation at 1070 nm), naturally occurring fluorophores such as NADH and flavins (e.g. FAD) can be efficiently excited [54]. This enables label-free imaging of endogenous biomolecules. Using this setup, we successfully imaged an outer epidermal cell layer of an onion at an excitation power of just 580 µW, which, to the best of our knowledge, is the lowest power reported for three-photon excitation of an autofluorescence signal [55,56]. This combination of deeper imaging, reduced phototoxicity, and high excitation efficiency highlights the advantages of our setup for advanced three-photon microscopy applications.

Remarkably, even at these low excitation powers, we were able to conduct advanced image analysis techniques such as the spectral phasor approach. This method provides valuable insights into the biochemical composition and spatial organization of the imaged sample. The ability to achieve such detailed spectral analysis at low excitation powers further highlights the versatility and efficiency of our system for a wide range of applications, including fluorescence lifetime imaging microscopy with direct sampling [57].

However, we also note some limitations of this approach. One concern is the potential for PMT saturation. At low repetition rates, the energy per pulse increases significantly to maintain sufficient signal levels, which can lead to fluorophore saturation and bleaching effects, especially in highly fluorescent regions of the sample. Similarly, the high instantaneous photon flux can saturate the PMT, compromising image quality. Additionally, while the average power at the sample is reduced, the high peak power per pulse may still cause localized photodamage or photobleaching, particularly in sensitive samples. Moreover, at lower repetition frequencies (e.g., used in this study 0.3 MHz), the pixel dwell time cannot be shortened to the same extent as at higher frequencies, leading to longer exposure of each pixel to high-intensity pulses, especially compared with resonant scanning systems [58]. This can increase the risks of localized photodamage or photobleaching. The longer scanning time can also pose practical challenges for users, especially when imaging large areas or dynamic samples, as it increases the acquisition time. These limitations prove that there is a need for careful optimization of the parameters of the excitation beam and the detector to balance the benefits of low-power imaging with potential risks.

## 5. Summary

This study presents a novel, all-fiber laser setup with a GMN amplifier, designed for advanced imaging applications. The fully fiber amplifier design (until the output compressor) provides compactness, robustness, and ease of alignment compared to free-space systems, making it practical for diverse experimental settings. The integration of a pulse picking unit allows the user to perform imaging at exceptionally low average power levels (even as low as 66 µW), which can lead to the minimization of photodamage and photobleaching. This is critical for long-term observation of living specimens and imaging fragile materials, such as thin films.

The system supports advanced imaging techniques, including the spectral phasor approach, even at low excitation power, showcasing its versatility. Its use of the GMN amplifier enables the efficient use of different multiphoton modalities, even the 3PEF, by enabling the use of longer wavelengths for deeper tissue penetration, reduced scattering, and enhanced excitation efficiency. Thanks to these features, the system is a valuable advancement for expanding the capabilities of multiphoton microscopy in diverse applications.

## Supplemental information

Supplement 1 Supplemental Document https://doi.org/10.6084/m9.figshare.28640027

## Data Availability

Data underlying the results presented in this paper are available in [42].

## Supplemental document

See Supplement 1 for supporting content.
