# Supplementary material for: Multiphoton microscopy at a microwatt level via gain-managed nonlinear amplification and pulse-picking
Source: Biomed Opt Express. 2025 Mar 27;16(4):1692–706. doi: 10.1364/BOE.557132 (PMC12047729; doi:10.1364/BOE.557132)
Supplement: Supplementary file 1 [file boe-16-4-1692-s001.pdf]

Supplemental Document

Biomedical Optics EXPRESS

Multiphoton microscopy at a microwatt level via gain-managed nonlinear amplification and pulse-picking: supplement

KATARZYNA KUNIO, GRZEGORZ SOBOŃ, AND JAKUB BOGUSŁAWSKI*

Laser & Fiber Electronics Group, Faculty of Electronics, Photonics and Microsystems, Wrocław University of Science and Technology, Wybrzeże Wyspiańskiego 27, 50-370 Wrocław, Poland
*jakub.boguslawski@pwr.edu.pl

This supplement published with Optica Publishing Group on 27 March 2025 by The Authors under the terms of the Creative Commons Attribution 4.0 License in the format provided by the authors and unedited. Further distribution of this work must maintain attribution to the author(s) and the published article's title, journal citation, and DOI.

Supplement DOI: https://doi.org/10.6084/m9.figshare.28640027

Parent Article DOI: https://doi.org/10.1364/BOE.557132

MULTIPHOTON MICROSCOPY AT A MICROWATT LEVEL VIA GAIN-MANAGED NONLINEAR AMPLIFICATION AND PULSE-PICKING: SUPPLEMENTAL DOCUMENT

Additional characterization of the laser system is presented in Fig. S1. Figure S1(a) shows the oscillator's fundamental beat note of radio frequency (RF) spectrum at 15.23 MHz with the SNR of 74 dB. The measurement confirms a very good stability of mode-locking operation. Figures S1(b) – (c) present the characterization of the pulse at the gain-managed nonlinear (GMN) amplifier's output (i.e. after propagation through the pulse picking unit and the GMN amplifier). The RF spectrum shown in Fig. S1(b) displays overall good stability. The period between pulses shown in the pulse train in Fig. S1(c) is equal to 66 ns, which corresponds to the fundamental repetition frequency of 15.23 MHz. The amplitude modulation of both the RF spectrum and pulse train was observed at the output of the oscillator, as shown in [1], and it propagates to the output of the amplifier. We note that this effect does not influence imaging performance as multiple pulses are averaged per pixel.

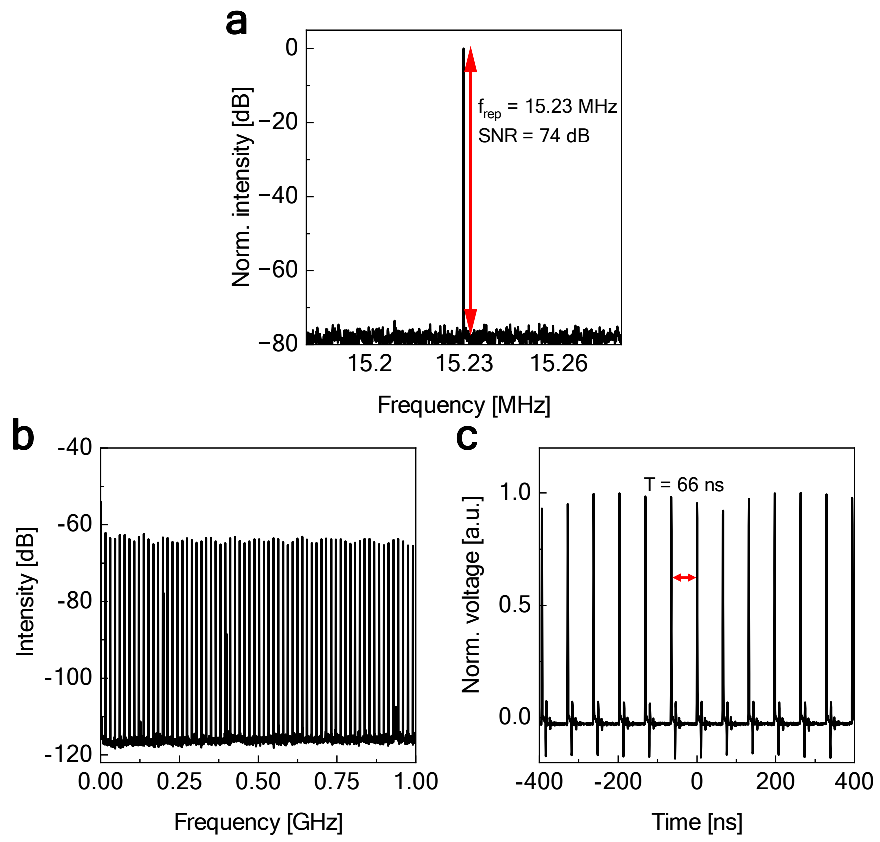

Figure S1. (a) The fundamental beat note of the oscillator's RF spectrum. Characterization of the pulse at the GMN amplifier's output: (b) the RF spectrum, (c) oscilloscope trace of the pulse train.

References

1. K. Kunio, J. Bogusławski, and G. Soboń, "Efficient multiphoton microscopy with picosecond laser pulses," Opt. Lett. 49, 4597 (2024).
